# You are only as safe as your riskiest contact: Effective COVID-19 vaccine distribution using local network information

**DOI:** 10.1016/j.pmedr.2022.101787

**Published:** 2022-04-05

**Authors:** Alec M. McGail, Scott L. Feld, John A. Schneider

**Affiliations:** a Cornell University, Ithaca NY, USA; b Purdue University, Lafayette IN, USA; c The University of Chicago, Chicago IL, USA

**Keywords:** Targeted vaccination, COVID-19, Friendship paradox, Network epidemiology

## Abstract

When vaccines are limited, prior research has suggested it is most protective to distribute vaccines to the most central individuals – those who are most likely to spread the disease. But surveying the population’s social network is a costly and time-consuming endeavour, often not completed before vaccination must begin. This paper validates a local targeting method for distributing vaccines. That is, ask randomly chosen individuals to nominate for vaccination the person they are in contact with who has the most disease-spreading contacts. Even better, ask that person to nominate the next person for vaccination, and so on. To validate this approach, we simulate the spread of COVID-19 along empirical contact networks collected in two high schools, in the United States and France, pre-COVID. These weighted networks are built by recording whenever students are in close spatial proximity and facing one another. We show here that nomination of most popular contacts performs significantly better than random vaccination, and on par with strategies which assume a full survey of the population. These results are robust over a range of realistic disease-spread parameters, as well as a larger synthetic contact network of 3000 individuals.

## Highlights

• Using simulation to evaluate nomination of most popular contacts for vaccination.
• Simulating spread of COVID-19 across two contact networks among high-schoolers.
• Targeting in this way can reduce spread to the susceptible population by 20% or more.
• Results are robust in a synthetic network replicating spread in a small town.
• Results are robust across a wide range of infectiousness, and mistaken nomination.

## 1 Introduction

**Fig. 1:**
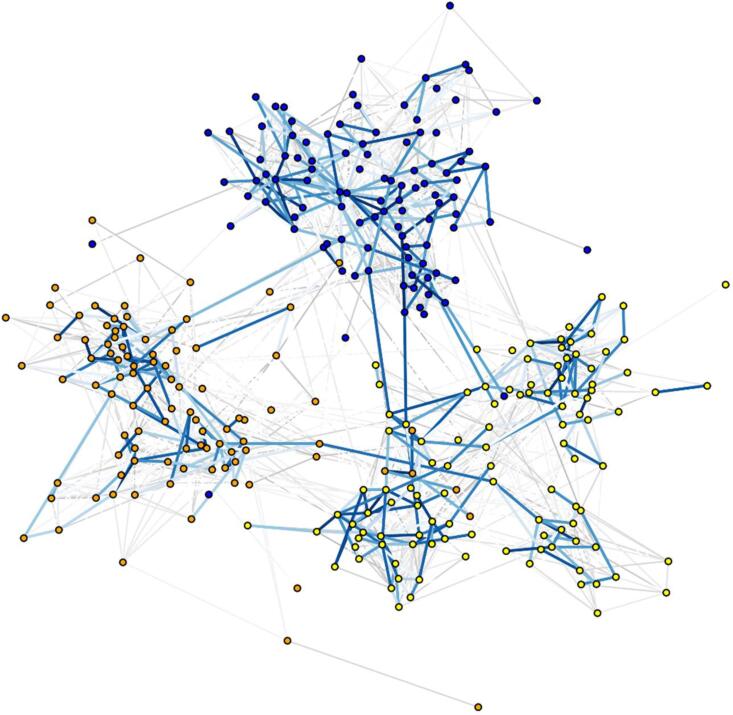
HS-1. Contact network between students in a French lycee, one Tuesday in 2013. Nodes are coloured by their grade level.

**Fig. 2:**
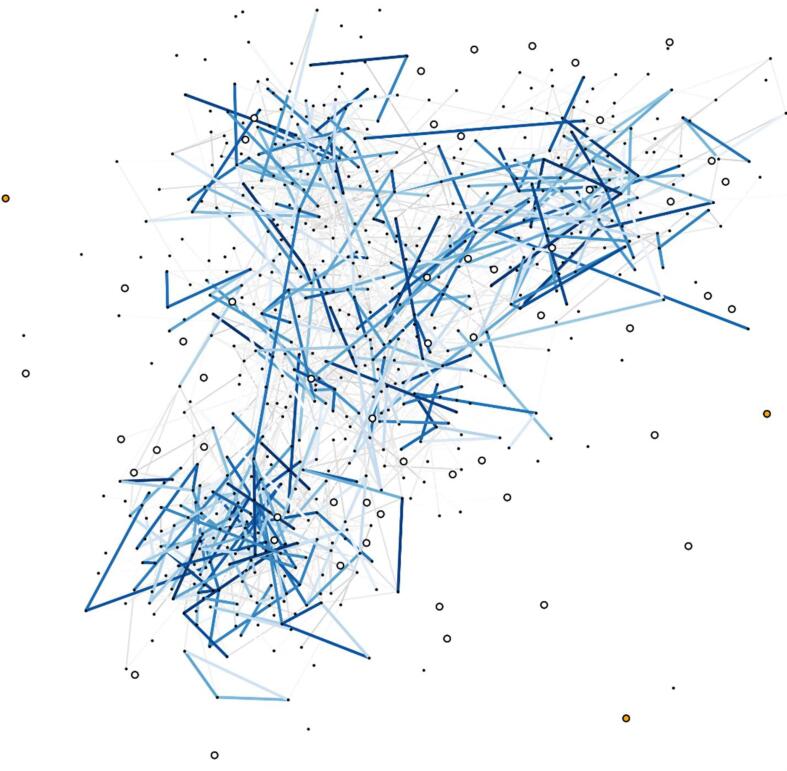
HS-2. The contact network between 656 students, 56 staff, 73 teachers, and 5 others in a U.S. high school, one Thursday in 2010. Nodes with a black border are non-students, and are essentially disconnected from the student contact network.

**Fig. 3:**
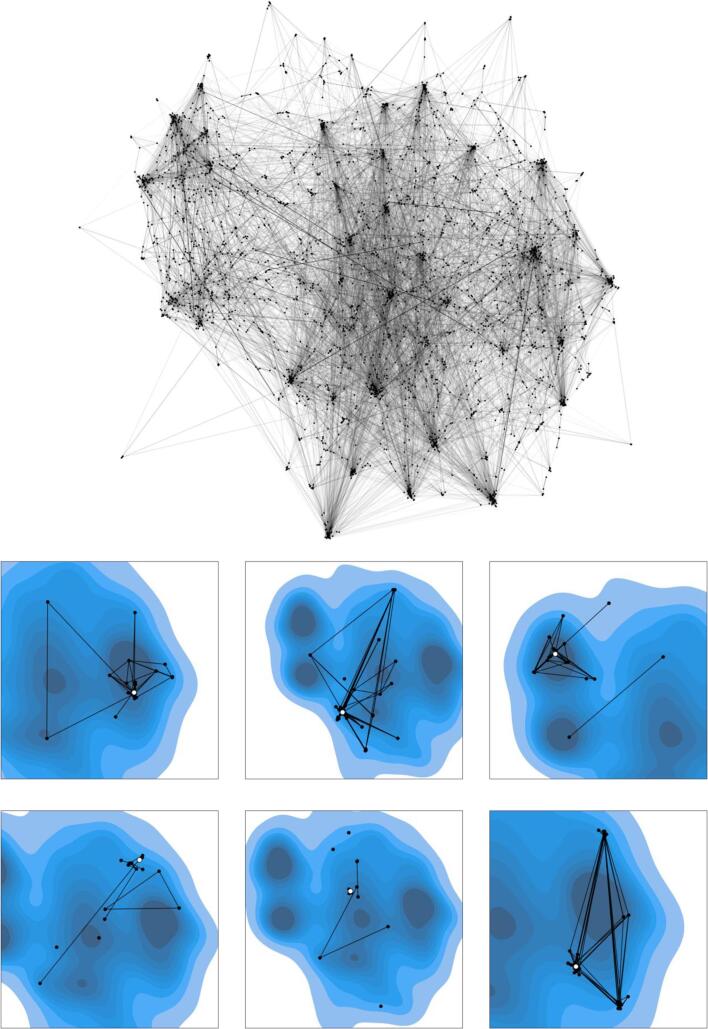
Synthetic contact network of 3000 individuals, clustered within age-groups and riveted together into family units.

**Fig. 4:**
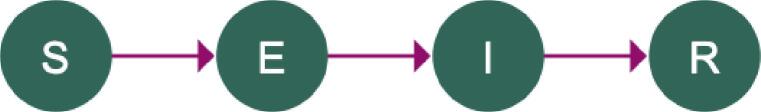
Illustration of disease spread model used in this paper. Exposure (E) is predicated on contact with an infected (I) individual. Transitions E → I and I → R are drawn from exponential distributions.

Although viable vaccines for COVID-19 are now widely in use, accessibility to the vaccine is progressing slowly through the world. As of this writing (April 20, 2021), 40% of the U.S. population have received at least one dose of the vaccine, almost 50% of the U.K. and 62% of Israelis. However, only 6.5% of the world’s population has had at least one dose and less than 1% in Africa. In light of this stark reality, and in response to the possible need for distributing new vaccines to fight new strains of this virus or others, we should find ways to improve the effectiveness of the limited vaccines a community, school, or nation has available (Fig. 1, Fig. 2, Fig. 3, Fig. 4).

**Fig. 5:**
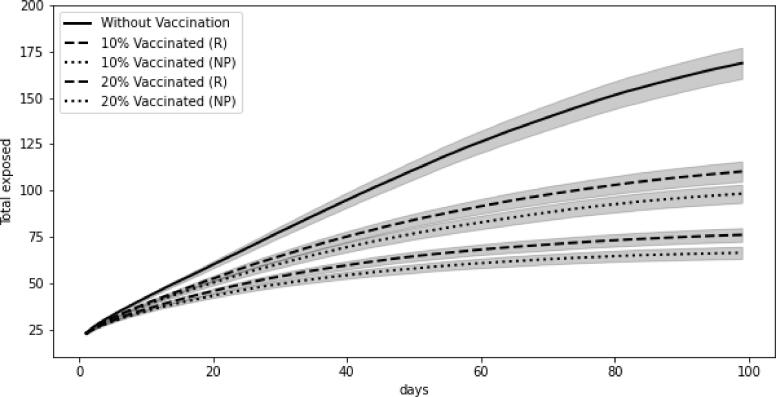
The average number of exposures over the course of 100 days in HS-2, with E[R0] = 2.5 and initially infecting 20 individuals. The gray strip around each trend represents the 95% confidence interval, $\pm 1.96*\frac{s}{\sqrt{N}}$ where s is the sample standard deviation and N is the number of simulations, 500.

The current dominant method for vaccine prioritisation is to first vaccinate those most vulnerable, then front-line workers most likely to be exposed to the disease, working eventually towards herd immunity at around 70% of the population vaccinated. This overall strategy for targeting has been recently shown through simulation to be optimal in avoiding hospitalization and death (Jahn et al., 2021). To reduce total infections and deaths we can employ a more nuanced targeting strategy which aims at those who are most likely to spread the disease. This methodology does not necessarily supplant the prioritisation just mentioned but may be used to complement it. For example, within a nursing home, local nomination strategies could be used to choose who to vaccinate first and may yield important transmitters as opposed to an age or comorbidity approach. In addition, after these highest risk groups are vaccinated a targeted approach could be used to vaccinate the population at large.

Prior work has shown that individuals most central in the disease-spread network are the most important targets for vaccines (Dezső and Barabási, 2002, Jia et al., 2020, Pastor-Satorras and Vespignani, 2002), and even in the specific context of COVID-19 (Jadidi et al., 2020). Nunner et al. (2022) recently demonstrated that targeting occupational categories as a proxy for connectedness in a contact network is quite effective. Some work has pointed to the importance of decentralised methods for nominating those who should be vaccinated (Cohen et al., 2003, Hébert-Dufresne et al., 2013, Holme, 2004, Ke and Yi, 2006, Lee et al., 2012, Taghavian et al., 2017). One compelling method chooses a random individual and nominates for vaccination a random of their contacts. This “random nomination strategy” relies on the Friendship Paradox, the fact that these random contacts will be more connected than random individuals are (Feld, 1991). This strategy has been shown to be more effective than random vaccination (Wang et al., 2016, Manzo and van de Rijt, 2020 for COVID in particular). A related strategy asks individuals to recall who they interacted with most recently, relying on the recurring nature of interactions. Lee et al. (2012) have shown that this method outperforms random nomination.

In this paper we suggest the *nomination of most popular contacts* (NP) as a practical and effective method. In this method administrators choose an individual at random and ask them to nominate a contact of theirs who has disease-spreading contact with the most people. Although similar strategies have been proposed and evaluated in the physics literature (Holme, 2004, Ke and Yi, 2006, Wang et al., 2016), they have been ignored by epidemiologists and policymakers. One possible reason, which leads to the central contribution of this paper, is that the models they use, and the networks on which they evaluate these strategies, are simplifications at best. Holme (2004) finds that chained nomination of most popular contacts, NP(c) in this paper, is the most effective local targeting strategy of those he analysed, but he does not test this using realistic contact networks, nor perturbing the model of disease spread or (of course) calibrating this model to COVID-19 in particular.

**Fig. 6:**
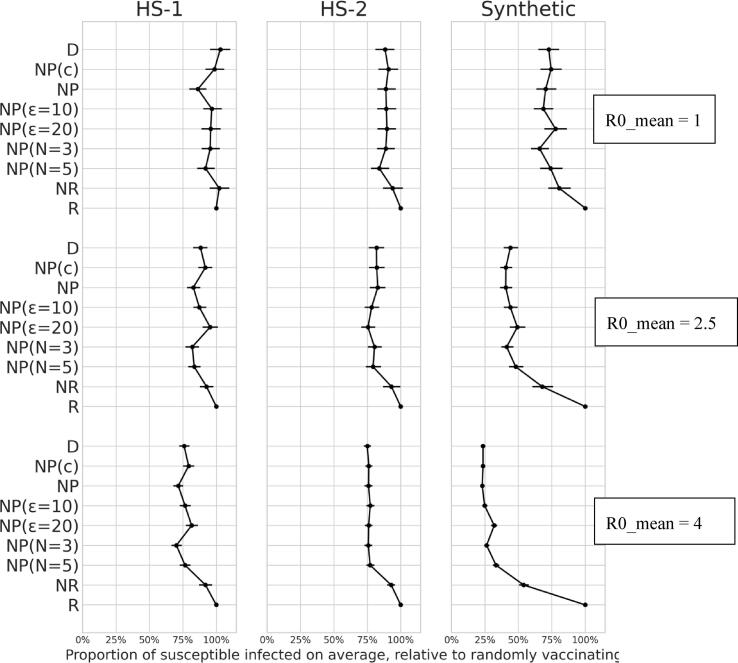
Effectiveness by infectiousness. This figure presents the effectiveness of degree-based nomination (D), variations of popularity-based nomination (NP), and random nomination (NR), with different average infectiousness (R0_mean). In all scenarios we assume 20% of the population is vaccinated, and 20 individuals are infected at the start. The measure reported on the x-axis is what proportion of the susceptible population is infected, relative to random vaccination (R).

**Fig. 7:**
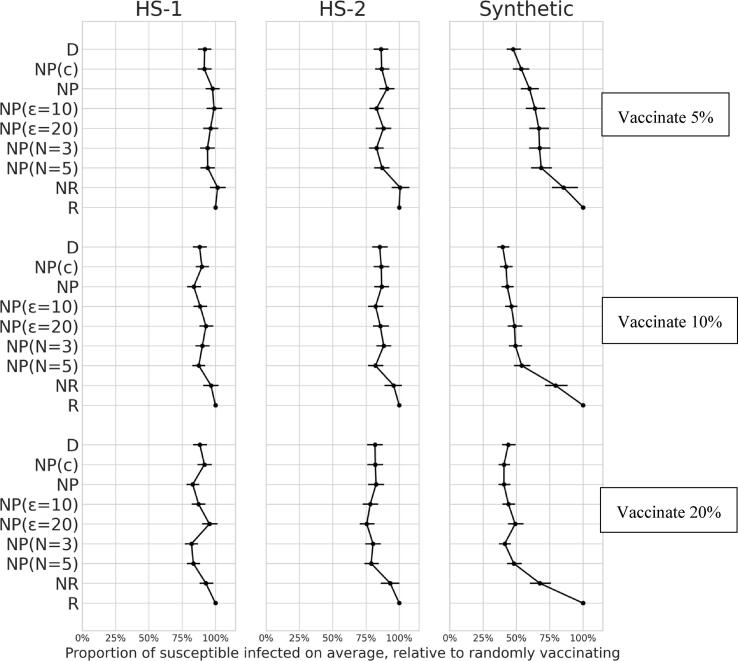
Effectiveness by proportion vaccinated. This figure presents the effectiveness of degree-based nomination (D), variations of popularity-based nomination (NP), and random nomination (NR), with different proportions of the population vaccinated. In all scenarios we assume 20% of the population is vaccinated, and 20 individuals are infected at the start. The measure reported on the x-axis is what proportion of the susceptible population is infected, relative to random vaccination (R).

This paper evaluates the strategy using more realistic simulations, which simulate the spread of COVID-19 on contact networks measured from physical interactions in a real-world setting, and presents results in a digestible form, in the hopes of spurring renewed policy interest in decentralised targeting strategies for vaccines. Our analyses show a marked robustness of the effectiveness of nomination of most popular contacts over a wide range of disease spread models over three contact networks, and with some loosening of the assumption that individuals can accurately nominate their most contacted contact. The DATA and METHODS sections describe the contact networks, targeting strategies, and simulation methodologies. We conclude with Fig. 6 and Fig. 7, which detail the relative effectiveness of the vaccine strategies across combinations of network and model of disease spread.

## 2 Data

### 2.1 Empirical contact networks

Epidemiological models often assume homogeneous mixing, where an infected individual has equal probability of infecting anyone else in the population. And when epidemiologists employ a networked approach, they often use an unweighted network, where contact either exists or does not between each pair of interactants, with no variation. Both these assumptions are patently false (Bioglio et al., 2016), and variation in contact proves instrumental to accurate modelling of disease spread (Manzo and van de Rijt, 2020, Stehlé et al., 2011). In this paper we include the more practical and differentiated structure of the contact network of two high schools as they operated pre-COVID. We focus on high-school students as they are the most likely to not follow public health or other authority recommendations around social distancing and mask wearing. Furthermore, in the United States the rate of infection is twice as high in those aged 12–17 compared to 5–11-year-olds (Leeb et al., 2020).

In these two studies (*HS-1*, Mastrandrea et al., 2015, and *HS-2*, Salathé et al., 2010), students wore battery-powered Bluetooth transmitters / receivers which exchange packets of information whenever students are in close physical proximity with each other. The signals do not travel as far through solid objects, including students’ bodies. They most reliably communicate when students are face-to-face and within a distance of approximately 6 feet (in *HS-1*) and approximately 3 feet (in *HS-2*). The sensors have been designed to reliably determine colocation in each 20s interval, resulting in an extremely high-resolution contact network. These temporal contact networks have been extensively and independently evaluated for the purpose of studying respiratory diseases whose main vector of transmission is across such short distances. As such they are an ideal source for plausible simulations of the spread of COVID-19.

The physical and social structures of these two schools yield social networks that are similar in some ways, and different in others. The French lycée (*HS-1*) is split into three grades, each of which are split into three classes. Students mostly interact within their own class, but in the hallways, during lunch, and before and after school, we see many more cross-class contacts, especially within grade. Some students act as a bridge between classes and there is strong age homophily. The American high school (*HS-2*) has many of the same characteristics, but split across four grade levels, and with much more between-class interactions. HS-2 is also more than double the size. These structural elements, amongst others which I have not noted, are embedded as features of the HS-1 and HS-2 networks upon which we simulate the spread of COVID-19 in this paper. In all, the differences between these two schools and the two sensor methodologies offer strong robustness checks to the results we present.

### 2.2 Synthetic network of a town

The third contact network we use in this paper represents a small town of 3000 residents and was generated procedurally using SEIRS+ (McGee et al., 2021). The algorithm (an adaptation of FARZ) reproduces the clustering and degree distribution observed on average in the United States. This network also reproduces the age distribution of United States citizens, and the differential probability of contact between those of different age groups. Within each of the four age groups a community structure is generated, representing primary schools, secondary schools, workplaces, and elderly community structures. Average degree by age group was matched to an empirical measurement of contact networks in the United States (Mossong et al., 2008). Individuals from different age groups are then grouped into households, matching the distribution of household sizes and the household age demographics of the United States.

## 3 Methods

The contact network and a realistic model of disease spread together constitute a complete understanding of the spread of a disease. But both are heterogeneous across local contexts, and to some extent unmeasurable. How exactly COVID-19 spreads depends on many factors, and our understanding of these factors is still incomplete. For example, the probability that an infected individual spreads the disease to another person in one day is very hard to measure and depends on a variety of factors. Masking, ventilation, the physical arrangement of a space, these all contribute to reducing the probability of contagion. These propensities are also affected by individual attributes such as age, and a social context’s relation to the outside environment. Different vaccination strategies have differential relative effectiveness depending on the number of vaccines administered, and who is available for vaccination at all, which in turn depends on institutional, logistic, and individual psychological factors. Individuals differ in how COVID-19 affects them and their subsequent infectiousness, and the scope of this variability and its relationship with network position are not entirely known. In response to the existing knowledge and extant uncertainty of how COVID-19 spreads, we test a range of modifications to the central nomination strategy, in addition to perturbing average infectiousness, the variation in infectiousness across the population, the percent vaccinated, the number of initial infections, all in addition to testing in the context of the three contact networks described above. The following subsections detail the vaccine targeting strategies and simulation methodology we consider.

### 3.1 Vaccine targeting strategies

**R – Random** Each person is equally likely to be vaccinated.

**D – Degree –** First determine the number of contacts each person has (their so-called degree). Choose the N people who have the highest degree.

**NR – Random nomination** – 1) Choose a person at random. 2) From their unvaccinated contacts choose a person to be vaccinated at random. 3) Repeat steps 1 and 2 until N individuals are vaccinated. As shown by Feld (1991), randomly nominated individuals are on average more central than random individuals (R), and this method is a common benchmark for decentralized strategies.

**NP – Nomination of most Popular contacts –** Same as *Random Nomination (NR)*, except people nominate their unvaccinated contact who is in contact with the most other people.

**NP(ε) – Nomination of most Popular contacts with Gaussian error –** Same as *Nomination of most Popular contacts (NP)*, except individuals will estimate their contacts’ number of contacts with error which is normally distributed with standard deviation ε.

**NP(N) – Nomination amongst top N most Popular contacts –** Same as *Nomination of most Popular contacts (NP)*, except individuals will nominate randomly from their top N highest degree unvaccinated contacts.

**(c) – Chained nomination** – Each nomination strategy listed above has an accompanying “chained” version. Instead of fetching a new random person for each new nomination, we have the most recently vaccinated person make the nomination. Whenever they cannot (all their friends have been vaccinated), or at the beginning when no people have been vaccinated, we start a new chain with a random person.

For example, NP(c), *chained nomination of most popular contacts* begins by choosing a person to be vaccinated at random. They then choose their unvaccinated contact who has the most contacts to be vaccinated second. This person then nominates a third, and so on, until the most recently vaccinated person can no longer nominate a person. The process then begins again from a randomly chosen unvaccinated person, continuing until we have run out of vaccines or people.

### 3.2 Simulation methodology

We use a stochastic SEIR (susceptible, exposed, infected, removed) model for disease spread, following the model contributed and compiled by McGee et al. (2021). In this model, a susceptible person is exposed (E) to COVID-19 by one of their infected (I) contacts. The exposure occurs at a randomly chosen time, drawn from an exponential distribution with mean depending on the infectiousness of the infected person, the susceptibility of the exposed person, and the amount of in-person contact they share. Once exposed, an individual will after some time move into the infected (I) state where they can expose others, and some time later will move into the recovered (R) state, no longer infectious or susceptible to infection. The latent period between the exposed and infected states and the recovery time are also drawn from exponential distributions, with expected means which are somewhat different for each person. In the case of the synthetic network, these parameters are tuned to match what we know of their age-dependence in the case of COVID-19. For full details on the distributions we used for these parameters, see the online supplement.

The daily probability of spread for COVID-19 has been measured to be anywhere from less than 0.05 to 0.2 on average for those who come into contact in that day (Carcione et al., 2020, Jiang et al., 2020, Mwalili et al., 2020), corresponding to a wide range of R_0_ anywhere from near 1.0 to upwards of 5. This does not only reflect an uncertainty of the “true” transmissibility of COVID. Instead it reflects the heterogeneity of this average transmissibility across different contexts. A typical estimate in the literature is 2.5 (e.g. as used in Manzo and van der Rijt), but in this paper we vary R_0_ along this entire range, assessing to what extent differences in average transmissibility may change the overall results. We generate R_0_ for each individual based on a gamma distribution. For the central models in this paper we assume a relatively low coefficient of variation CV[R_0_] = 0.2, which describes the variation in personal R_0_ across the population, although the supplement checks robustness with respect to this parameter. Note also that we assume that those aged 0–19 are half as susceptible to infection as those aged 20 +.

To begin the simulation, according to one of the targeting methods detailed in the previous section, we assume that some group of individuals had been vaccinated at the start of the simulation, and are not at all susceptible to the infection. They are fully removed from the disease-spread network. Then we randomly infect some number of unvaccinated individuals. We then run the simulation for 100 days. The measure we use for the effectiveness of any given strategy is the total number of individuals who entered the exposed (E) state at any time in the 100 days. For each set of parameter values, we run 500 independent simulations, choosing again who to vaccinate and who to infect, in order to estimate accurately the properties of the distribution of total infections under these scenarios. We report uncertainty in our estimate of the true mean infected by the standard error. For uncertainty in the percent improvement over not vaccinating or random vaccination, we bootstrap from the sampled simulation results. We collect 10,000 samples with replacement, with sample size 500, and calculate the quantiles of the relevant ratios corresponding to a 95% CI.

Because HS-1 and HS-2 are empirically gathered contact datasets, we assume that the measured contact is the only contact on which disease may spread. However, the synthetic network generates strong contact ties according to what we know of institutional and family ties. This would leave out spread which occurs in public and interstitial spaces. And so for the synthetic network alone we assume a propensity of random spread to any other node in the network, in addition to the propensity of spread along network ties. This is constant throughout, set at 20% of an individual’s total disease-spread contact.

**Table:** 

	***Parameter Name***	***Tested Values***	***Description***
**E[R0]**	Average individuals’ infectiousness	1, **2.5**, 4	The expected number of additional infections in a completely connected population, given a seed infection.
**CV[R0]**	Coefficient of variation of individuals’ infectiousness	**0.2**, 0.8, 1.4, 2.0, 2.5	Parametrizes the influence of individual super-spreaders in the dynamics of the infection.
**S**	Starting Infections	5, 10, **20**	Number of infections at the start of the simulation.
**SV**	Percent vaccinated at the start of the simulation	5%, 10%, **20%**	Percent of the population vaccinated on the first day of the simulation.
**Net**	Network on which disease spreads.	HS-1, HS-2, Synthetic	These networks are described in **Data**.

## 4 Results

First we present the relative effectiveness of these vaccination strategies under one scenario. That is, with the average infectiousness R_0_ = 2.5 (with coefficient of variation = 0.2), and initially infecting 20 individuals. The average numbers of individuals infected in each context when we do not vaccinate at all, across 500 simulations, are 28.0 ± 0.6 of the 290 remaining susceptible in HS-1 (9.7%), 149.8 ± 3.3 of the 764 remaining susceptible in HS-2 (19.6%), and 267.6 ± 10.6 of the 2980 remaining susceptible in the synthetic network (9.0%). More than 25% of the population is infected in 0.2% (HS-1), 29.4% (HS-2), and 2.0% (the synthetic network) of simulations. Vaccinating 20% of the population randomly will decrease the average number of infections after 100 days by 22.8% (HS-1; 18.3% – 26.9%), 52.9% (HS-2; 49.5% – 55.7%), and 62.3% (the synthetic network; 57.4% – 66.4%). And crucially for the purposes of this paper, nomination of most popular contacts (NP) does significantly better than random vaccination. Vaccinating 20% of the population in this way decreases the average number of infections after 100 days by a further 17.1% (HS-1; 12.1% – 22.0%), 17.3% (HS-2; 11.2% – 22.7%), and 59.5% (the synthetic network; 54.3% – 63.6%), relative to random vaccination. In parentheses are listed 95% confidence intervals as described in the Methods section. Fig. 5 shows this central comparison as it evolves over the 100 days.

This relative effectiveness will be our metric throughout the rest of this section. That is, what is the improvement of the vaccination strategy over randomly vaccinating individuals. We bring forward two robustness checks to the relative effectiveness of the vaccination strategies, that of modifying E[R0] (the average infectiousness in the population), and the proportion of individuals who are vaccinated. The relative effectiveness of strategies for E[R0] = [1, 2.5, 4] are presented in Fig. 6, and for SV = [5%, 10%, 20%] in Fig. 7. When E[R0] is higher the difference between randomly vaccinating and nominating most popular contacts increases, especially in the synthetic network. Indeed, the most extreme benefit of NP is observed for the synthetic network with E[R0] = 4. This is also the circumstance with the highest average number of infected individuals overall. In this case approximately 75% additional susceptible individuals are saved from infection on average by incorporating NP over random vaccination.

For HS-2 the incorporation of error in nomination through the NP(N) and NP(ε) does not have any detectable effect across these simulations, whereas error does moderate the effectiveness of NP for HS-1 and the synthetic network. In HS-1 in particular, for E[R0] of 1 or 2.5, erroneous reporting nearly fully diluted the positive effects of NP. Yet these circumstances are also those with the lowest average number of infected individuals overall.

A variety of additional robustness checks we have performed are detailed in the supplement. For instance, inclusion of NP(N) and NP(ε) in Fig. 6 and Fig. 7 show that moderate error in nominating highly connected contacts will for the most part not derail the effectiveness of this strategy. We might then ask how much error would it take. Once individuals are nominating from their top 10 or more friends, we found little difference between NP(N) and NR in all circumstances. Likewise for random Gaussian error, once the standard deviation of this error is greater than 30 contacts, NP(ε) is indistinguishable from NR (see Fig. S6). We would expect these strategies to monotonically approach NR (as distinct from R), and attribute the minor deviations from this pattern to the stochasticity of simulations. We also varied the number of individuals initially infected, as well as the variation in individuals’ R0, and found no substantive differences (Figs. S4 and S5). In addition, the number of individuals initially infected, a proxy for the extent of outside infections introduced, does not affect the order of strategies, but shows that the more dire the threat from outside, the more effective are these targeted strategies relative to random vaccination, at least in the parameter ranges we consider here (Fig. S5). In addition, we varied CV[R0], as a proxy for wider variability in infectiousness *independent of* network position. With dramatic increase in this variability, there was not an appreciable increase in effectiveness of the targeting strategies relative to random vaccination (Fig. S7).

For a deeper look at all the realised runs of the simulation we use in this paper as well as the remainder of these robustness checks, or to extend these results to new empirical settings or differently specified models of disease spread or vaccination nomination, see the accompanying repository at https://www.github.com/amcgail/episim/. We also verified in this repository that the ordering of daily contact in HS-1 and HS-2 were irrelevant for the outcomes reported here through explicitly simulating these dynamics.

## 5 Conclusion and Future Work

This paper tested the relative effectiveness of a strategy for choosing how to allocate limited vaccines to maximise their effectiveness. That is, to choose a random person and have them nominate from their contacts the individual with the most contacts. This method is aimed at logistical feasibility, allowing the administrator to survey individuals as they receive vaccinations, needing just one survey response to administer the first vaccine. This strategy performs significantly better than randomly distributing vaccines, but also performs better than choosing random contacts of individuals, a classic decentralized targeting strategy, and often even better than simply targeting those with the highest degree, assuming we have a full survey of the population of interest. Concretely, the majority of reasonable parameter combinations showed better than a 20% reduction in infections amongst the susceptible compared to randomly vaccinating, on average across 500 simulations. One may object that individuals’ reports of the interaction profiles of their contacts may prove more erroneous than self-reports. Yet, through the inclusion of the NP(ε) and NP(N) strategies, we were able to show that moderate error in nomination does not cripple the effectiveness of the method.

This simulation is necessarily limited, not including all features of realistic COVID-19 spread. Future work can explore the inclusion of various competing strains of COVID-19, variation in the initial composition of individuals in terms of having or having had COVID-19, heterogeneity in the effectiveness of vaccination which is not total and wanes over time, the temporality of contact networks, and many other complexities. In addition, a separate paper could address the variation of effectiveness under different measures, as compared to total number infected with COVID-19 after 100 days as analysed in this paper. It is also possible that most central individuals may not be optimal targets in practice. The most central may differ from the general population in various other ways, which may correlate with their unwillingness to be vaccinated (as in the model of Wells et al., 2013), or their probability of already being immune to the disease. We do not in this paper address the concrete issues of implementation such as the right survey strategy to approximate this theoretical model, and leave this to future work. Such work could additionally investigate how the targeting strategy proposed here could make use of the personal relationships between interviewee and target, to mobilize interpersonal trust and communication to convince an individual to get vaccinated, along the same lines of respondent-driven sampling (Heckathorn, 1997).

## Ethics Approval

N/A. No students were infected in this simulation study.

## Data Availability Statement

In order to replicate what we present here, and to encourage extension of our methodology and results, all code and data used in this paper are available at https://www.github.com/amcgail/episim/.

## CRediT authorship contribution statement

**Alec M. McGail:** Conceptualization, Methodology, Software, Visualization, Formal analysis. **Scott L. Feld:** Conceptualization, Supervision. **John A. Schneider:** Conceptualization, Supervision.

## Declaration of Competing Interest

The authors declare that they have no known competing financial interests or personal relationships that could have appeared to influence the work reported in this paper.
