# Supplementary material for: You are only as safe as your riskiest contact: Effective COVID-19 vaccine distribution using local network information
Source: Prev Med Rep. 2022 Apr 5;27:101787. doi: 10.1016/j.pmedr.2022.101787 (PMC8979884; doi:10.1016/j.pmedr.2022.101787)
Supplement: Supplementary data 1 [file mmc1.docx]

# Detailed Network Descriptives

The first (*HS-1*) recorded proximity throughout the school day for 327 French high school students over the course of 5 days. Because some students were not present or participating on multiple of the 5 days, we consider only the interaction profile for Tuesday, assuming these interactions repeat each day of the simulation. The 310 students who were present on Tuesday saw an average of 16.6 contacts for at least 20 seconds. The most was 43 contacts, and the least only 1. The pair who interacted most were together for a full 3.94 hours that day, but the mean pairwise contact time in the day was only 6.13 minutes. Students spent 1.70 hours on average facing another person in this way. The most social individual interacted with others for 8.11 hours on Tuesday.

The second high school (*HS-2*) was collected in a single day, recording proximity for 656 students, 56 staff, 73 teachers, and 5 others (784 total participants). Participants saw an average of 96.9 contacts for at least 20 seconds. The most was 248 contacts, and the least only 1. The pair who interacted most were together for a full 2.79 hours that day, but the mean pairwise contact time was only 0.87 minutes. Students spent 2.27 hours per day on average facing another person in this way. The most social individual interacted with others for 6.48 hours that day.

# Full Modelling Details

## Parameter Details

The time it takes for an infected individual to expose one of their contacts is drawn from an exponential distribution with parameter specific to the individual, as well as the amount of contact between individuals. That is, individuals are heterogeneous in their infectiousness once they reach the infectious state, and subsequent infections are stochastic. For each person we draw the expected number of new infections generated by their infection (individual R_0_) from a gamma distribution. The coefficient of variation is an important parameter for the individual R_0_ distribution in that it tunes the degree of superspreading in the heterogeneous transmissibility. Individuals also differ in the expected time it takes them to move between states. That is, in their latent period (between E and I), and their infectious period (between I and R). These transitions also happen in exponential random time.

Xin et al. (2021a) fit a model to available data from the Chinese context to estimate the latent period to be best fit as a gamma distribution with mean 5.5 (95% CI: 5.1–5.9). A meta-analysis of 72 studies of the incubation period of COVID-19 (Xin et al. 2021b) found estimates to vary widely with the majority falling between 5 and 10 days, and fit using a variety of distributions including the gamma. They estimate a pooled mean of 6.3 days. Ogata et al. (2022) found that the unvaccinated infected with the Delta strain had shorter incubation times than non-Delta cases in Japan, 3.7 vs. 5.0 days. In all simulations presented in this paper, the latent period was assumed to have a mean of 5.5 days and coefficient of variation of 0.6 with gamma distribution, and individuals are assumed to be infectious for a further 6.2 days on average, a combination of pre-symptomatic and symptomatic gamma distributions. This disregards the variability over time of infectiousness, considering an individual as either infectious or not, an approximation made for the purpose of computational tractability. The chosen distribution of period of infectiousness is based on extant research on transmissibility over time of the infected (see He et al. 2020, Meyerowitz et al. 2020).

These parameter values vary widely from model to model in the COVID-19 simulation literature (Xiang et al. 2021), and although we expect our results to be general with respect to these particular parameters, we have attempted in this paper to approximate the dynamics of COVID-19 in particular. The default distributions are given in Figures S1, S2, and S3 for HS-1, but they are similar for HS-2 and the Synthetic network. The individual R0 distribution shown in Figure 3 will depend on E[R0] and CV[R0], but has a similarly shaped distribution.

Figure S1. The distribution of pre-symptomatic period, (a)symptomatic period, and total infectious period in HS-1 simulations. The distributions are essentially identical for all other simulations run in this paper.


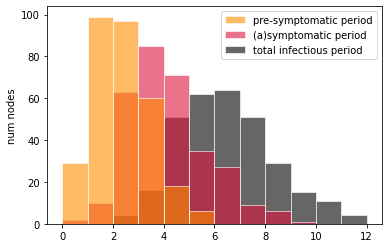


Figure S2. The distribution of latent period, pre-symptomatic period, and total incubation period in HS-1 simulations. The distributions are essentially identical for all other simulations run in this paper.


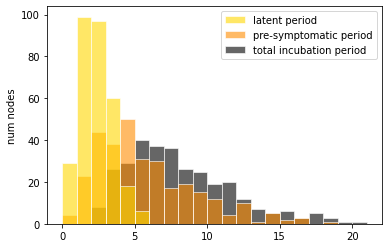


Figure S3. The distribution of individual R0 in HS-1 simulations, where E[R0] = 2.5 and CV[R0] = 0.2.


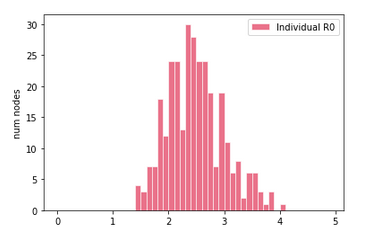


## Temporal Networks, Speedup and Slowdown

Prior work on temporal networks has found either a speed-up or slow-down of infection when the ordering and timing of contact, as against just the frequency, are taken into account (Karsai et al. 2011; Masuda & Holme 2013). Indeed, recent work has shown that simply rescheduling contact events in a hospital setting can dramatically reduce the spread of a highly infectious disease (Valdano et al. 2021). Yet because of the relatively slow spread of COVID-19 compared to the high frequency data available from both settings we analyse here, the intricacies of these real-time contact networks can be safely collapsed to daily contact frequencies, with state changes occurring on a daily basis, with no loss of realism (see the accompanying <http://github.com/amcgail/episim> repository for verification; Stehlé et al. 2011 for corroboration).

## Mapping of Contact Networks to Relative Infectiousness

We opt for a linear mapping of pairwise contact in HS-1 and HS-2 to the multiplier for pairwise infectiousness propensity. This amounts to mapping 15m of face-to-face contact in one day to a multiplier of 1. One hour amounts to four times this multiplier, and so-on.

# Additional Robustness Checks

Figures S4 – S9 follow the same pattern as Figures 5 and 6 presented in the main paper, exploring separate robustness checks. Most are discussed in the body of the paper. S10 and S11 explore the effect of limits in vaccine effectiveness on substantive results.

Figure S4. This figure presents the effectiveness of degree-based nomination (D), variations of popularity-based nomination (NP), and random nomination (NR), with different variation in individual infectiousness CV[R0].


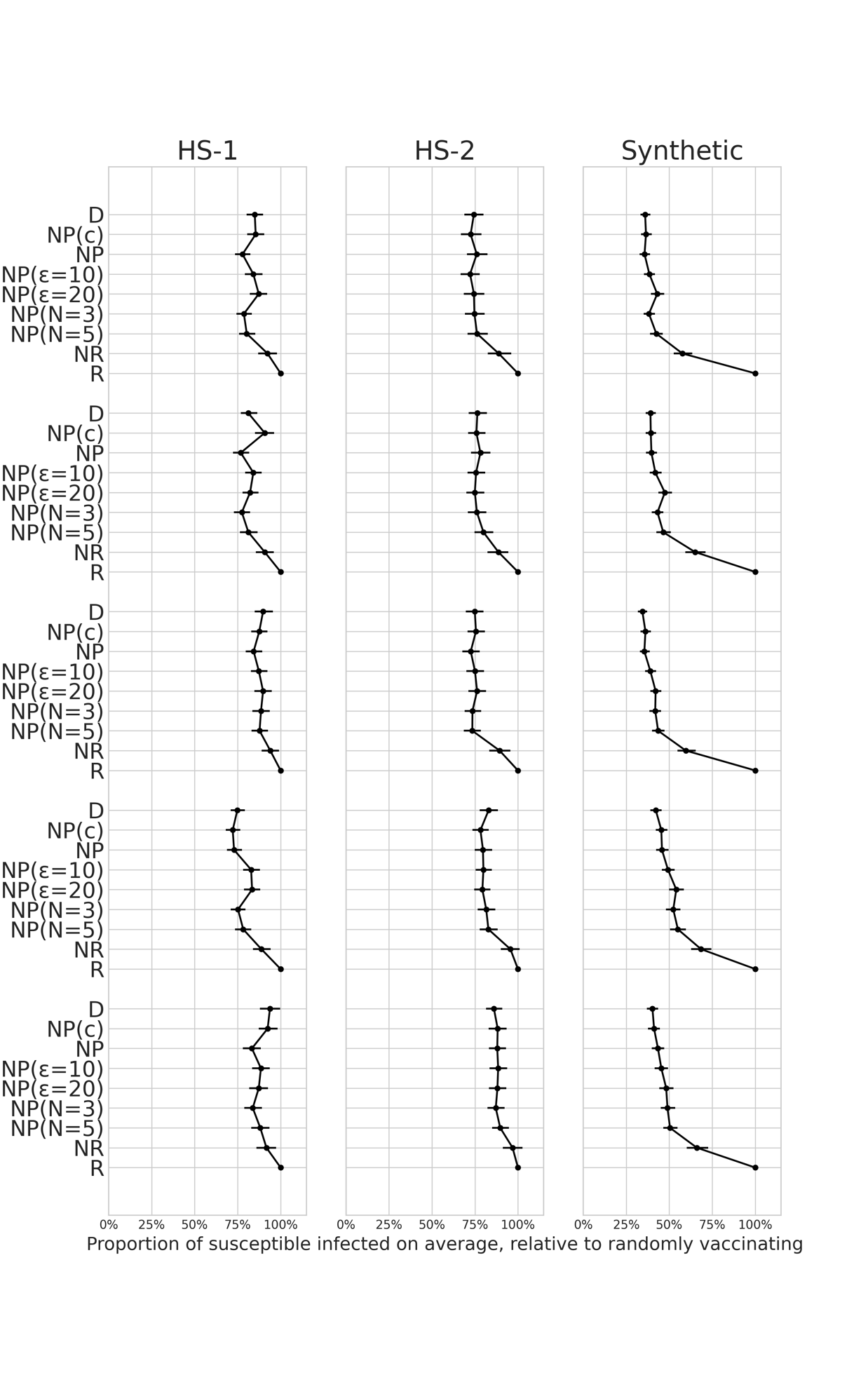


CV[R0] = 2.5

CV[R0] = 2.0

CV[R0] = 1.4

CV[R0] = 0.8

CV[R0] = 0.2

Figure S5. This figure presents the effectiveness of degree-based nomination (D), variations of popularity-based nomination (NP), and random nomination (NR), with different initial number of infections.


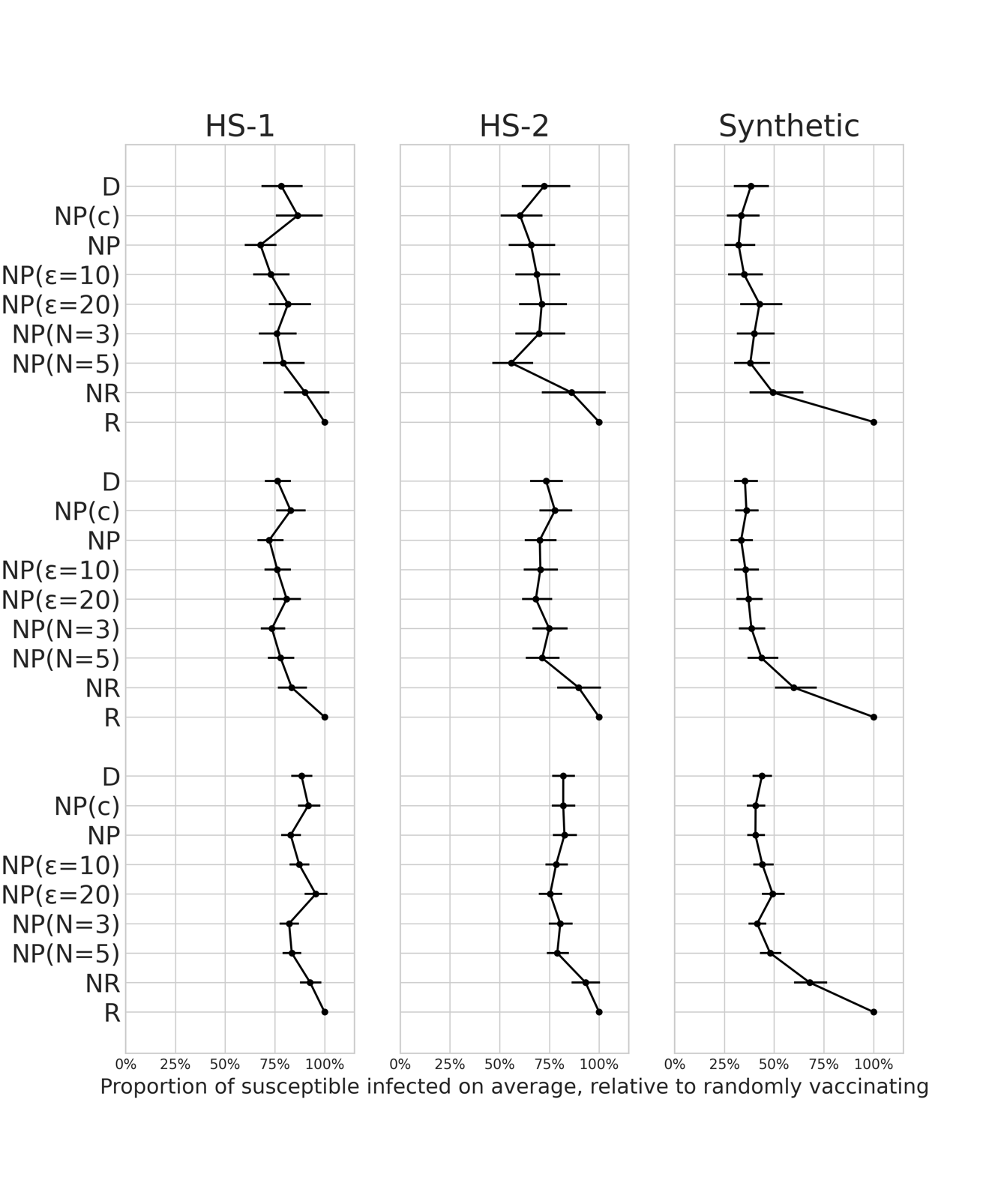


Infect 5 at start

Infect 10 at start

Infect 20 at start

Figure S6. Investigation of higher levels of random error in nomination. We see that for larger errors, the benefits of NP essentially disappear.


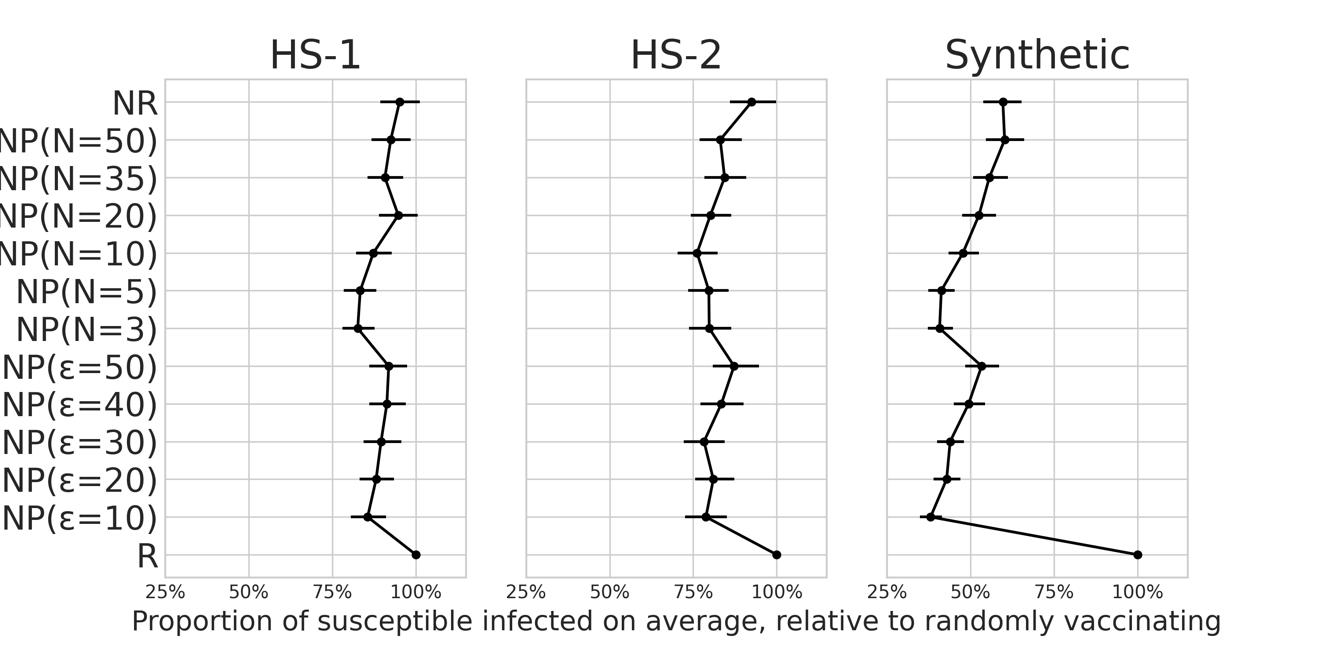


Figure S7. Investigation of the dependence of results on regeneration of individual-level attributes for each new simulation. “Single argset” produces these attributes once, as in the paper. “300 argsets” produces these attributes anew for each simulation. This mirrors Figure 5 in the text.


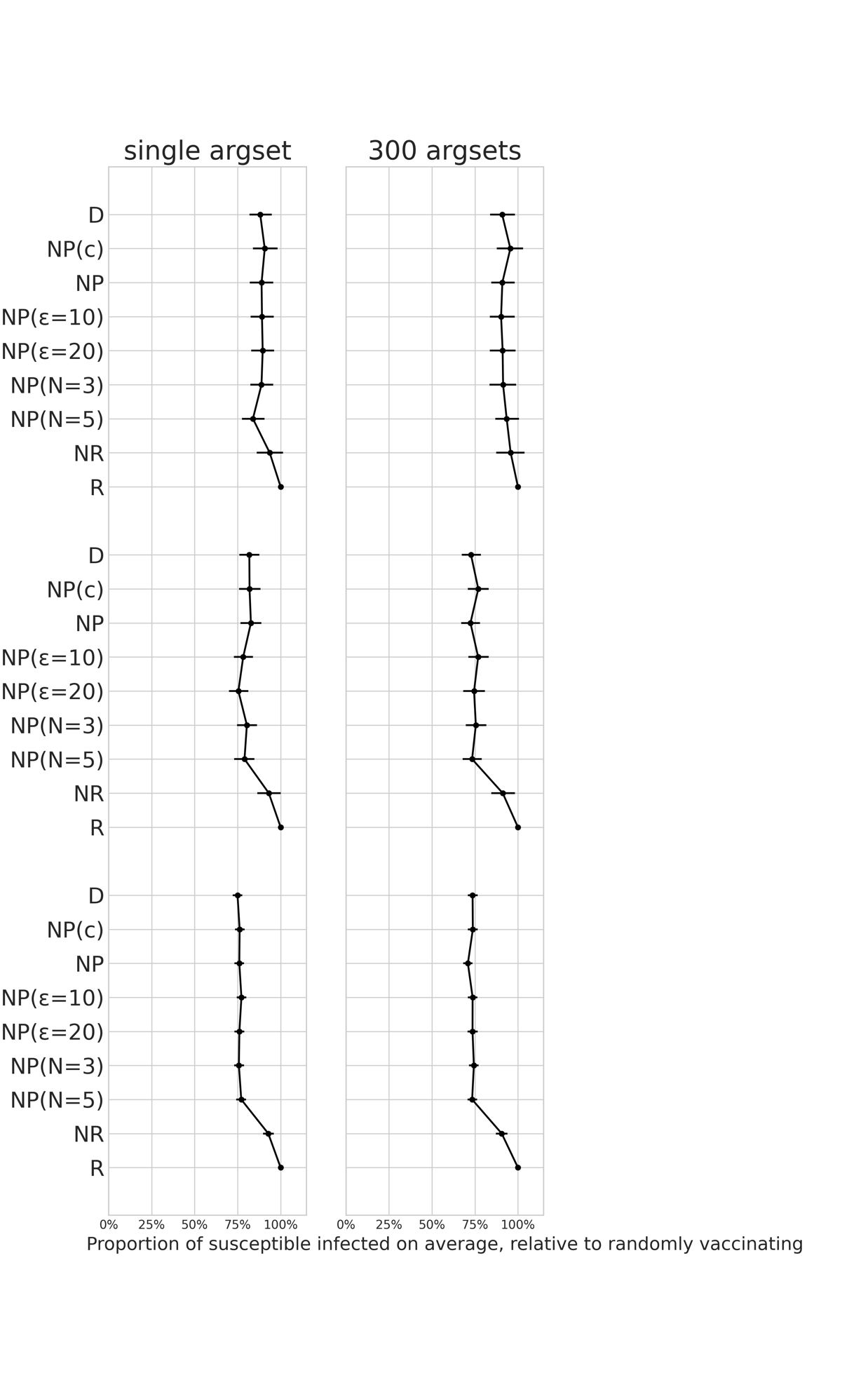


R0_mean = 4

R0_mean = 1

R0_mean = 2.5

Figure S8. Investigation of the dependence of results on regeneration of individual-level attributes for each new simulation. “Single argset” produces these attributes once, as in the paper. “300 argsets” produces these attributes anew for each simulation. This mirrors Figure 4 in the text.


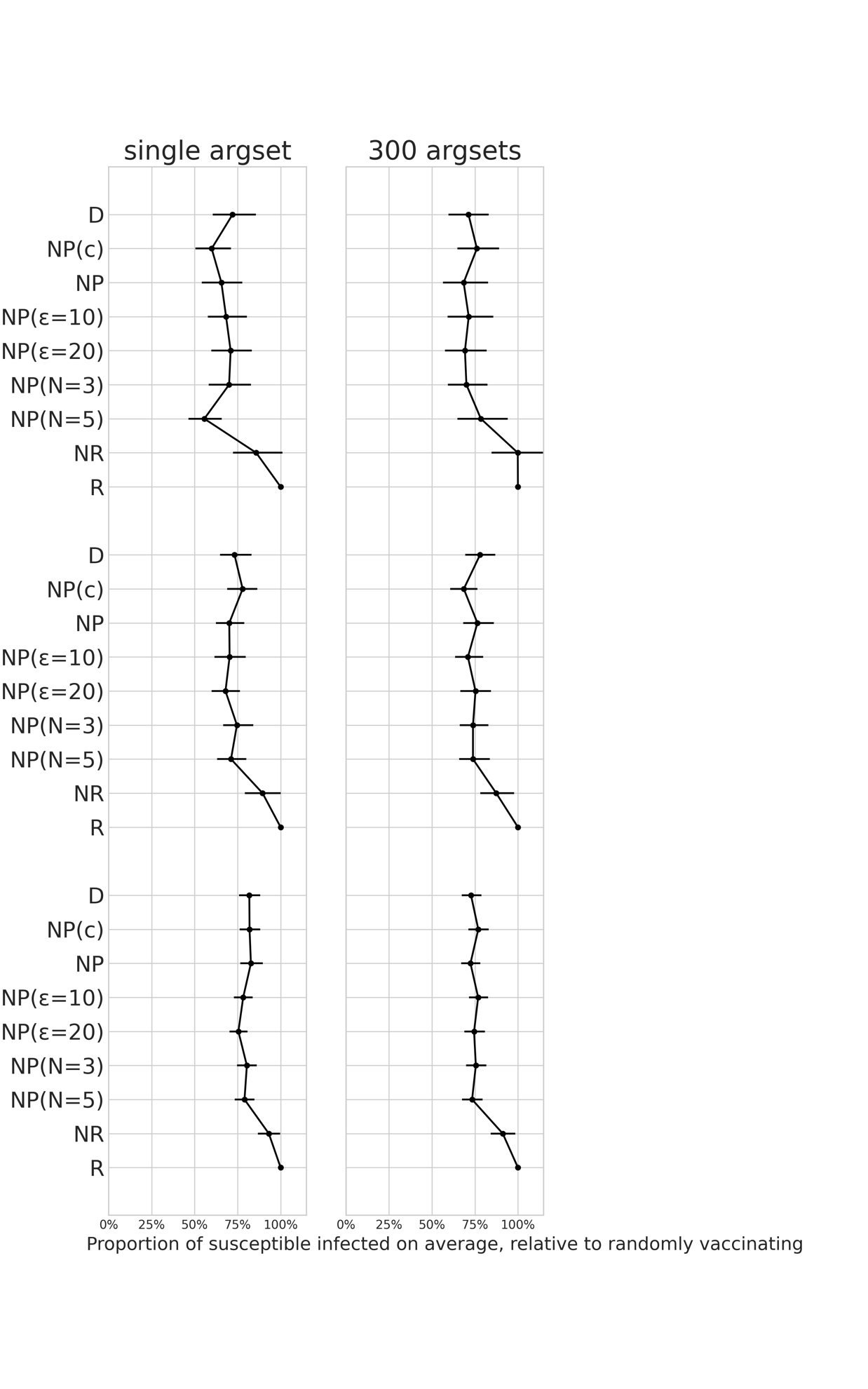


Infect 20 initially

Infect 5 initially

Infect 10 initially

Figure S9. Investigation of the dependence of results on regeneration of individual-level attributes for each new simulation. “Single argset” produces these attributes once, as in the paper. “300 argsets” produces these attributes anew for each simulation. This mirrors Figure 6 in the text.


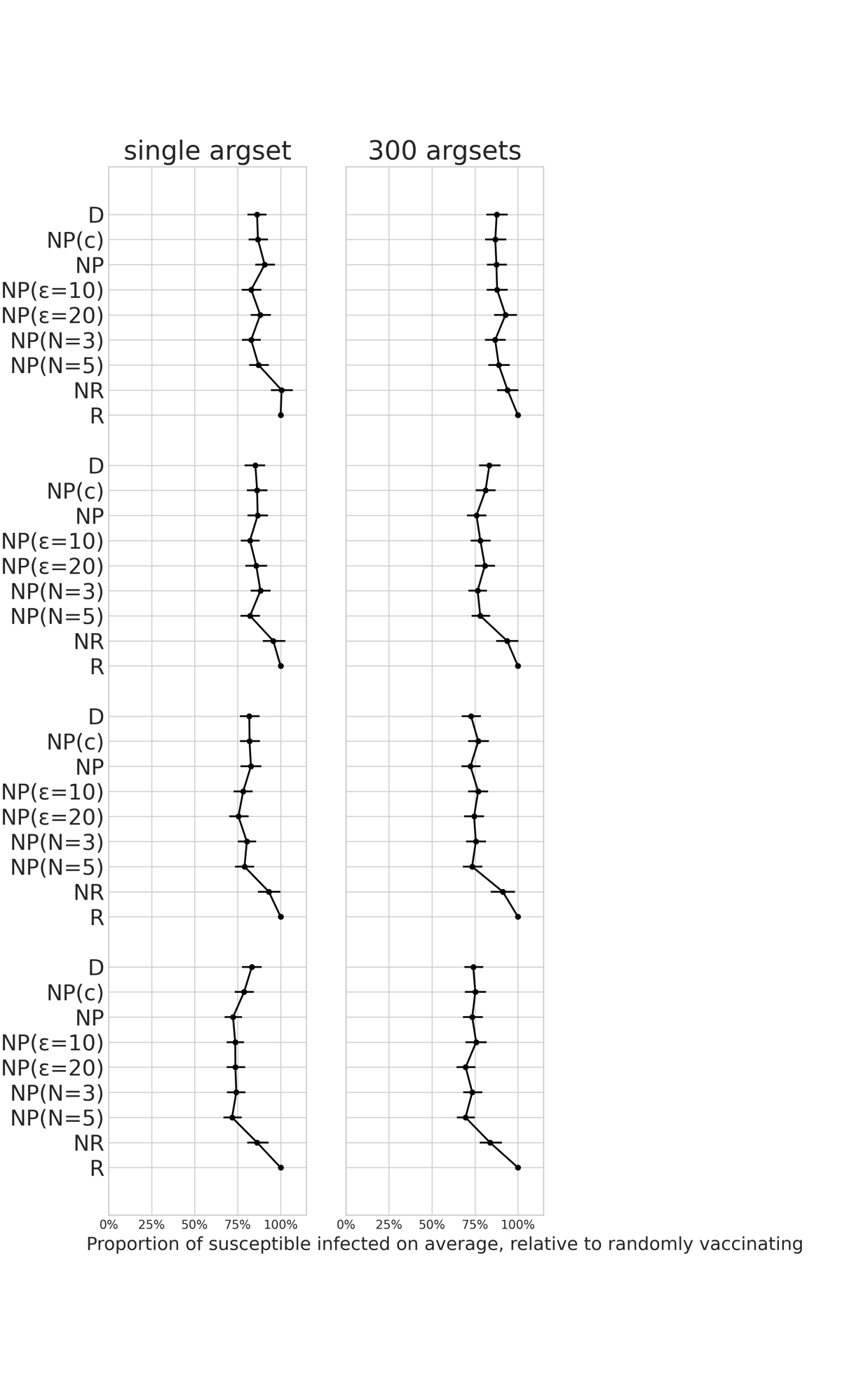


Vaccinate 50%

Vaccinate 20%

Vaccinate 10%

Vaccinate 5%

Figure S10. Shows how effectiveness relative to random vaccination varies by the effectiveness of vaccines in HS-1. 1000 simulations were run for these comparisons, in order to reduce error bars.


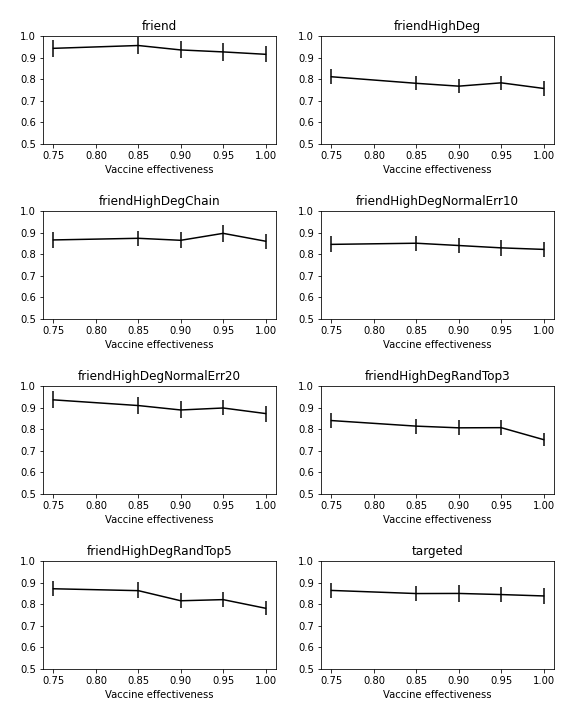


Figure S11. Shows how effectiveness relative to random vaccination varies by the effectiveness of vaccines in HS-2. 1000 simulations were run for these comparisons, in order to reduce error bars.


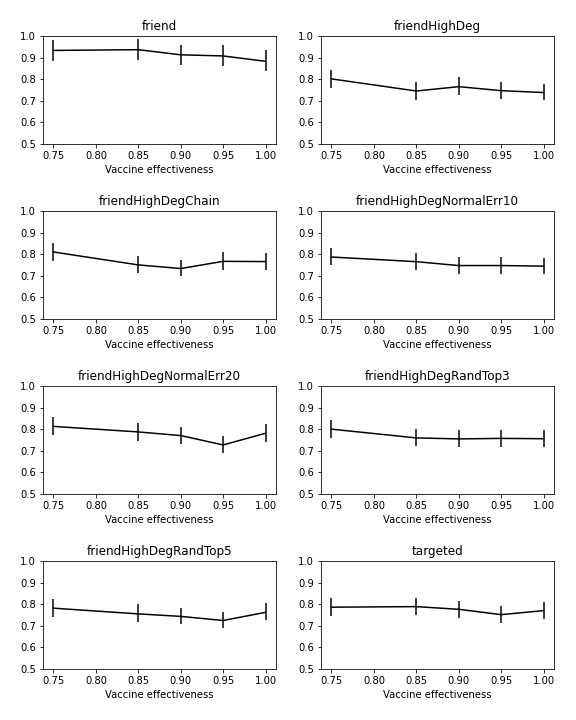


# The Overlap of Methods of Nomination

It is interesting and instructive to investigate to what extent different methods ended up nominating the same individuals for vaccination. Figure S12, Figure S13, and Figure S14 show the overlap between methods for *HS-1*, *HS-2*, and the *Synthetic* network, respectively. To produce these, we had each method choose 10% of *HS-1* to vaccinate, and repeated this 500 times. Each square then shows the average percent of overlap over these 500 runs. The diagonal represents the average overlap between subsequent runs. Degree-based nomination is the only deterministic method, and so we see the D/D square is equal to 100%.

Figure S12. The overlap of each pair of targeting methods evaluated in the text, for *HS-1*.


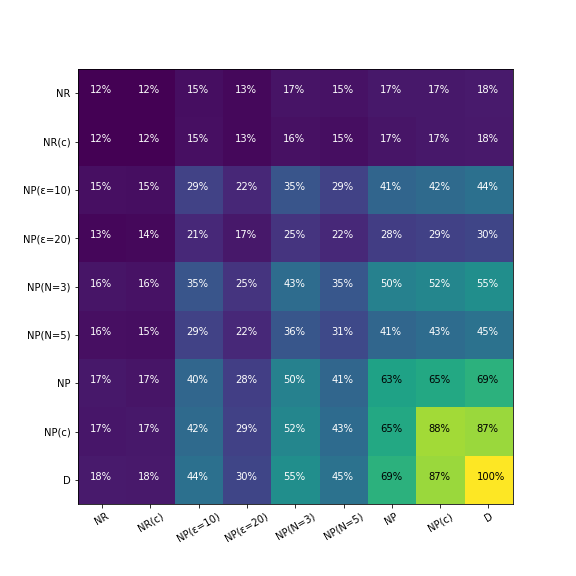


Figure S13. The overlap of each pair of targeting methods evaluated in the text, for *HS-2*.


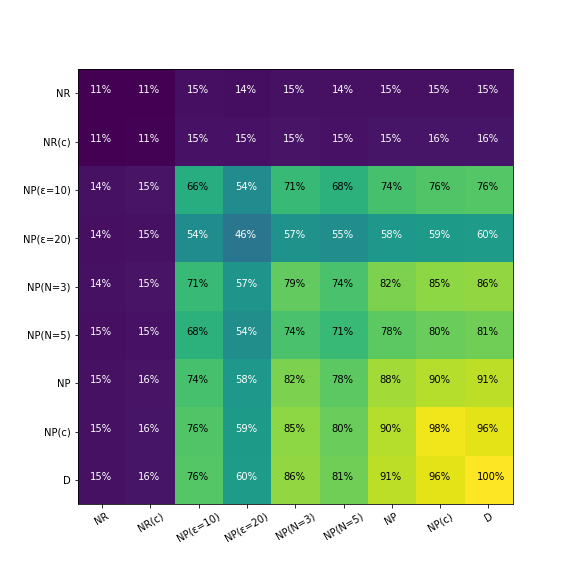


Figure S14. The overlap of each pair of targeting methods evaluated in the text, for the *Synthetic* network.


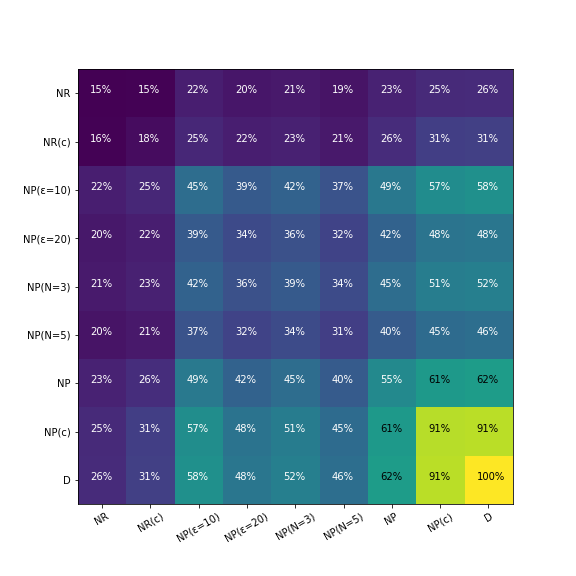


# References

He, X., Lau, E. H. Y., Wu, P., Deng, X., Wang, J., Hao, X., Lau, Y. C., Wong, J. Y., Guan, Y., Tan, X., Mo, X., Chen, Y., Liao, B., Chen, W., Hu, F., Zhang, Q., Zhong, M., Wu, Y., Zhao, L., Zhang, F., Cowling, B. J., Li, F., & Leung, G. M. (2020). Temporal dynamics in viral shedding and transmissibility of COVID-19. *Nature Medicine*, *26*(5), 672–675. <https://doi.org/10.1038/s41591-020-0869-5>

Karsai, M., Kivelä, M., Pan, R. K., Kaski, K., Kertész, J., Barabási, A. L., & Saramäki, J. (2011). Small but slow world: How network topology and burstiness slow down spreading. *Physical Review E - Statistical, Nonlinear, and Soft Matter Physics*, *83*(2), 1–4. <https://doi.org/10.1103/PhysRevE.83.025102>

Masuda, N., & Holme, P. (2013). Predicting and controlling infectious disease epidemics using temporal networks. *F1000Prime Reports*, *5*(March). <https://doi.org/10.12703/P5-6>

Meyerowitz, E. A., Richterman, A., Gandhi, R. T., & Sax, P. E. (2021). Transmission of sars-cov-2: A review of viral, host, and environmental factors. In *Annals of Internal Medicine* (Vol. 174, Issue 1, pp. 69–79). American College of Physicians. <https://doi.org/10.7326/M20-5008>

Ogata, T., Tanaka, H., Irie, F., Hirayama, A., & Takahashi, Y. (2022). Shorter Incubation Period among Unvaccinated Delta Variant Coronavirus Disease 2019 Patients in Japan. *International Journal of Environmental Research and Public Health*, *19*(3). <https://doi.org/10.3390/ijerph19031127>

Stehlé, J., Voirin, N., Barrat, A., Cattuto, C., Colizza, V., Isella, L., Régis, C., Pinton, J., Khanafer, N., Broeck, V. Den, & Vanhems, P. (2011). Simulation of an SEIR infectious disease model on the dynamic contact network of conference attendees. *BMC Medicine*, *9*(87), 1–15.

Valdano, E., Poletto, C., Boëlle, P. Y., & Colizza, V. (2021). Reorganization of nurse scheduling reduces the risk of healthcare associated infections. *Scientific Reports*, *11*(1), 1–18. <https://doi.org/10.1038/s41598-021-86637-w>

Xiang, Y., Jia, Y., Chen, L., Guo, L., Shu, B., & Long, E. (2021). COVID-19 epidemic prediction and the impact of public health interventions: A review of COVID-19 epidemic models. *Infectious Disease Modelling*, *6*, 324–342. <https://doi.org/10.1016/j.idm.2021.01.001>

Xin, H., Li, Y., Wu, P., Li, Z., Lau, E. H. Y., Qin, Y., Wang, L., Cowling, B. J., Tsang, T. K., & Li, Z. (2021). Estimating the Latent Period of Coronavirus Disease 2019 (COVID-19). *Clinical Infectious Diseases*. <https://doi.org/10.1093/cid/ciab746>

Xin, H., Wong, J. Y., Murphy, C., Yeung, A., Taslim Ali, S., Wu, P., & Cowling, B. J. (2021). The Incubation Period Distribution of Coronavirus Disease 2019: A Systematic Review and Meta-analysis. *In* *Clinical Infectious Diseases* (Vol. 73, Issue 12, pp. 2344–2352). Oxford University Press. <https://doi.org/10.1093/cid/ciab501>
